# The phylogeography of *Indoplanorbis exustus *(Gastropoda: Planorbidae) in Asia

**DOI:** 10.1186/1756-3305-3-57

**Published:** 2010-07-05

**Authors:** Liang Liu, Mohammed MH Mondal, Mohamed A Idris, Hakim S Lokman, PRV Jayanthe Rajapakse, Fadjar Satrija, Jose L Diaz, E Suchart Upatham, Stephen W Attwood

**Affiliations:** 1 State Key Laboratory of Biotherapy, West China Hospital, West China Medical School, Sichuan University, Chengdu 610041, China; 2 Department of Parasitology, Faculty of Veterinary Science, Bangladesh Agricultural University, Mymensingh 2202, Bangladesh; 3 Department of Microbiology and Immunology, College of Medicine, Sultan Qaboos University, Oman; 4 Infectious Diseases Research Centre, IMR, Jalan Pahang, 50588 Kuala Lumpur, Malaysia; 5 Department of Veterinary Pathobiology, Faculty of Veterinary Medicine and Animal Science, University of Peradeniya, Peradeniya 20400, Sri Lanka; 6 Department of Animal Diseases and Veterinary Public Health, Faculty of Veterinary Medicine, Bogor Agricultural University (IPB), Jl. Agathis-Kampus IPB Darmaga, Bogor 16680, Indonesia; 7 Veterinary Inspection Board, Vitas, Tondo, Metro Manila, Philippines; 8 Department of Biology, Faculty of Science, Mahidol University, Bangkok, Thailand; 9 Department of Medical Science, Faculty of Science, Burapha University, Bangsaen, Chonburi, Thailand; 10 Department of Zoology, The Natural History Museum, London, UK

## Abstract

**Background:**

The freshwater snail *Indoplanorbis exustus *is found across India, Southeast Asia, central Asia (Afghanistan), Arabia and Africa. *Indoplanorbis *is of economic importance in that it is responsible for the transmission of several species of the genus *Schistosoma *which infect cattle and cause reduced livestock productivity. The snail is also of medical importance as a source of cercarial dermatitis among rural workers, particularly in India. In spite of its long history and wide geographical range, it is thought that *Indoplanorbis *includes only a single species. The aims of the present study were to date the radiation of *Indoplanorbis *across Asia so that the factors involved in its dispersal in the region could be tested, to reveal potential historical biogeographical events shaping the phylogeny of the snail, and to look for signs that *I. exustus *might be polyphyletic.

**Results:**

The results indicated a radiation beginning in the late Miocene with a divergence of an ancestral bulinine lineage into Assam and peninsular India clades. A Southeast Asian clade diverged from the peninsular India clade late-Pliocene; this clade then radiated at a much more rapid pace to colonize all of the sampled range of *Indoplanorbis *in the mid-Pleistocene.

**Conclusions:**

The phylogenetic depth of divergences between the Indian clades and Southeast Asian clades, together with habitat and parasitological differences suggest that *I. exustus *may comprise more than one species. The timescale estimated for the radiation suggests that the dispersal to Arabia and to Southeast Asia was facilitated by palaeogeographical events and climate change, and did not require human involvement. Further samples from Afghanistan, Africa and western India are required to refine the phylogeographical hypothesis and to include the African Recent dispersal.

## Background

### The medical importance of *Indoplanorbis*

The freshwater snail *Indoplanorbis exustus *(Deshayes, 1834) (Planorbidae: Bulininae) is the sole member of its genus and is widely distributed across the tropics as an important intermediate host for several trematode parasites (Trematoda: Digenea). *I. exustus *is best known as the intermediate host responsible for the transmision of *Schistosoma nasale *Rao, 1933 and *Schistosoma spindale *(Montgomery, 1906), as well as other trematodes such as *Echinostoma spp. *and some spirorchids (Digenea: Spirorchiidae)[1,2]. A third species of *Schistosoma, Schistosoma indicum *(Montgomery, 1906) is also transmitted by *I. exustus. *Although other snails have been implicated in transmission of these three *Schistosoma spp. *(e.g., *Lymnaea luteola, S. indicum *and *S. nasale *and *Lymnaea acuminata*, *S. nasale *and *S. spindale*), *I. exustus *is the most important host for *S. nasale *and *S. spindale*, as well as for *S. indicum *in certain regions. Indeed *I. exustus *may be the sole natural intermediate host for these three *Schistosoma *species on the Indian sub-continent[3]. The three *Schistosoma *species are parasites of Artiodactyla, in particular of buffaloes, cows, goats pigs and sheep. Two of the species cause intestinal schistosomiasis, whilst *S. nasale *inhabits blood vessels of the nasal mucosa and causes "snoring disease" in cattle[4]. Surveillance for cattle schistosomiasis is generally inadequate and the literature is limited, but some idea of the problem can be gained from past small scale studies. Surveys in Sri Lanka revealed a prevalence of *S. spindale *of 31.2% (of 901 cattle)[5], whilst in Bangladesh a similarly high prevalence of 36% has been reported[6]. More recently, in Kerala South India, prevalences have been reported of up to 57.3% in cattle, 50% in buffalo and 4.7% in goats[3].

In addition to causing disease in cattle, *I. exustus *has been implicated in outbeaks of cercarial dermatitis in human populations in India[7,8], Laos[9], Malaysia[10] and Thailand[11,12]. Cercarial dermatitis results from the cutaneous allergic reaction in people exposed to larval schistosomes (cercariae) shed by infected snails into freshwater bodies such as lakes, ponds, and paddy fields. The cercariae cause pruritis and papular eruptions, with often severe secondary infections, as they attempt to infect a non-permissive definitive host and die in the skin[13].

*Indoplanorbis exustus *(see Additional file 1) is a common snail across Southeast Asia and the Indian sub-continent, where it often acts as intermediate host for *S. spindale. *The snail is also found in the Middle East (Oman and Socotra) and Nigeria and the Ivory Coast; these findings were attributed by Brandt[14] to recent introductions by human activities (Brandt's view has been frequently cited in the literature on *Indoplanorbis*[15-17]). In view of the wide geographical range of this snail and its importance as a host for several species of *Schistosoma*, there is a need to understand the phylogenetics and dispersal history of *Indoplanorbis*. In contrast to Asia, the well documented appearance of the snail in Africa (e.g., Nigeria[18] and Ivory Coast[19]) and more recently (2002) in the Lesser Antilles[16], is almost certainly the result of introductions through human activities over the last 50-100 years.

*Indoplanorbis exustus *is a hermaphroditic invasive snail species with high fecundity. Within one year of introduction the snail is able to colonize habitats with well established populations of other pulmonate and prosobranch snails[16]. The snail requires a water temperature in excess of 15°C for maturation. At the optimum temperature of 30°C each snail can lay up to 800 eggs[20]. The capacity for self-fertilization and high fecundity probably underlies the invasive potential of the species. The snail is found in small ponds, pools and less commonly in rice paddy fields. The snail may also occur in semi-permanent pools formed in flooded areas of fields, where it can survive the dry season buried in mud. Consequently, dispersal may occur in clumps of mud adhered to the bodies of cattle or across water in flotsam (vegetation mats), and possibly also attached to migratory birds (although this has not been observed for *I. exustus*). In Northeast Thailand, where *I. exustus *is very common, the annual rainfall is 1541 mm. In Assam, in the region where the snails were sampled for this study, the annual rainfall is much higher at around 4200 mm (data recorded 1982-2002)[21]. The current rainfall in Arabia is 340 mm per year; however, in Plio-Pleistocene humid periods this has risen to 630 mm or more[22]. It has been recognized that it is the length and severity of the dry season that influences vegetation type (savanna, rain forest, monsoon forest, etc) rather than the annual rainfall[23]. The rainfall in the dry season of Assam is approximately 250 mm, whereas that in Northeast Thailand is only 30-40 mm[21]. These observations suggest that the Assam/Bangladesh populations are subject to different ecological conditions than those of the Southeast Asian mainland (SEAM); these observations support the need to question the monophyly of *Indoplanorbis*. The invasive nature and ecological tolerance of the snails adds to their importance in veterinary and medical science.

### The historical biogeography of *Indoplanorbis*

The Lymnaeae and Planorbidae appear to share a common ancestor in the Permian (*c.a. *250 Ma (Mega annum or million years ago)) fossil record[24]. Fossil Planorbinae and Bulininae are known from the mid to upper Cretaceous of Africa and India[25]. The Tethys sea separated Africa and Laurasia until 10-5 Ma[26]. Consequently, Meier-Brook 1984[27] adopted an African (Gondwanan) origin for *Indoplanorbis *with rafting to Asia since the Cretaceous on the northward migrating Indian craton; this author also considered a Europe to Southwest Asia tract or an Africa to South India dispersal. Morgan et al. 2002 attributed the occurrence of *Indoplanorbis *in India to colonization (from Africa) via the Middle East land connection[28]. Similarly, Attwood et al. 2007 described a Sinai-Levant dispersal tract, from Africa to central Asia, for *Schistosoma spindale *during the early Pleistocene[29]. Clearly the two different dispersal mechanisms imply very different chronologies; the Gondwanan vicariance hypothesis implies that proto-*Indoplanorbis *has been present in India since the late Eocene (35 Ma; India: Asia collision[30]), whereas dispersal via the Sinai-Levant suggests a Plio-Pleistocene arrival.

The assumption that the *Indoplanorbis *lineage has been represented on the Indian craton since the Cretaceous is difficult justify in palaeogeographic terms. There would have been numerous opportunities for dispersal of Gondwanan taxa off India and into Asia throughout the Cenozoic. For example, during the middle Eocene (45 Ma) the northern corner of greater India came into contact with western Indonesia and subsequently with Sumatra and Myanmar[30]. Even prior to the true (hard) collison of India with Asia (i.e., between the Indian craton and the Lhasa block at 35 Ma), faunal exchange was possible via volcanic arc terranes (some forming exposed plateaus) in the oceanic crust subducting at the collision zone (*c.a. *130 Ma onwards)[31]. Thus the absence of a fossil record for *Indoplanorbis *or bulinine taxa from the Southeast Asian Palaeocene or Eocene argues against a Gonwanan-India rafting hypothesis for *Indoplanorbis *and favors a post-Miocene Sinai-Levant dispersal tract to India.

Earlier studies on *I. exustus *provide some DNA sequence data (cox1[32,33], nuclear 18S rRNA gene[32,33], mitochondrial (mt) 16S rRNA gene[32], and nuclear 28S rRNA gene[28]); however, these studies considered only one population, and in most cases did not report the location of the collecting site. In view of this, no population phylogenetic study had been possible prior to the present work. Collection site details were reported for one data set[33], which was recorded as being sampled from Loei Province northern Thailand[34]; these data were for the 18S gene and *cox*1, but did not include the 16S gene and were geographically close to the sample from Phitsanulok taken in the present study. Consequently, the Loei data were not added to the present analysis.

### Aims of the study

The palaeogeographic, fossil and biogeographical dispersal data described above imply a Miocene origin for the radiation of *Indoplanorbis *across Asia. Under such a model, colonization of India could have been followed almost immediately, by radiation across Southeast Asia through land bridges created by low eustatic sea levels during Pleistocene glacial excursions. The aim of this study was therefore to collect DNA sequence data for populations of the snails across the known range. These data could then be used to address three main questions. Firstly, the timescale for the dispersal and diversification of these snails across Asia; the time of arrival of taxa in various geographical regions is important because it sheds light on how long different host:parasite assemblages have been in contact and facilitates studies on phylogenetic tracking or co-evolution. Secondly, the work aimed to determine the historical events (climatic, tectonic or palaeogeogaphical) that may have influenced the phylogeography of these taxa. Thirdly, the taxonomic status of *Indoplanorbis *was considered. It seems improbable that only a single species of the genus has arisen if its radiation dates from the Miocene.

In order to answer the above questions, a population phylogeny for *Indoplanorbis *was first estimated and then used to elucidate major dispersal tracts and confirm the taxonomy of the genus (i.e., that only one species has arisen). Molecular clock methods were then used to date the radiation of these taxa so that the cladogenic events and dispersal tracts inferred could be related to any major historical environmental changes.

## Results

### Sequence data

Of the 15 taxa included in the analysis (see Table 1), 9 distinct haplotypes were found at the mitochondrial (mt) *rrn*L locus and 11 at the mt *cox*1 locus; there were 13 distinct haplotypes in the combined data set, with the taxa from Laos, Luzon and Thailand (Phitsanulok) being indistinguishable. The aligned *cox*1 data comprised 619 bps and the *rrn*L data 454 sites (396 bps without alignment gaps); the total aligned data set was therefore 1073 sites. The PTP-test indicated that the data showed significantly more structure than randomized data (*P *= 0.0002). The ILD-test was also significant, indicating that the two data partitions (*cox*1 and *rrn*L) should be modeled separately (*P *= 0.0203). Tests for deviations from neutrality were not significant: *cox*1 Fu * Li's D* -0.87029 (*P *> 0.10), F* -1.10963 (*P *> 0.10); *rrn*L D* -1.29227 (*P *> 0.10), F* -1.58492 (*P *> 0.10), all calculations were based on total number of mutations. Tajima's D: *cox*1 -1.23388 (*P *> 0.10); *rrn*L -1.63067 (*P *> 0.05). The test of Xia et al.[35] suggested that there were no significant levels of substitution saturation at either locus (ISS < ISS.C, *P *< 0.0001, a lack of statistical significance here would imply a poor phylogenetic signal).

**Table 1:** Locations and dates of samples used in the study

Country	Locality	Coordinates	Collection date	Accession number: *cox*1
*Indoplanorbis exustus*				***rrn*L**

Assam	Dibrugarh	27°30'08.2";94°57'06.7"	20/06/2002	[GenBank:GU451744] [GenBank:GU451726]

Bangladesh	Mymensingh	24°43'33.4";90°25'32.7"	12/07/2003	[GenBank:GU451745] [GenBank:GU451727]

Borneo (Malaysia)	Bintulu	02°52'31.0"; 112°50'19.0"	29/05/2002	[GenBank:GU451746] [GenBank:GU451728]

Indonesia	Bogor (West Java)	-6°27'44.5";106°42'25.1"	19/03/2005	[GenBank:GU451747] [GenBank:GU451729]

Laos	Xang	19°10'15.0";102°13'30.0"	14/05/2005	[GenBank:GU451750] [GenBank:GU451751]

Malaysia (West)	Kampang Pelegong	05°17'11.0";100°27'29.0"	26/05/2004	[GenBank:GU451738] [GenBank:GU451731]

Nepal	Janakpur	26°48'03.6";85°56'27.9"	12/04/2003	[GenBank:GU451739] [GenBank:GU451732]

Oman1	Wadi Bani Khaled	22°37'02.2"; 59°05'36.8"	25/11/04	[GenBank:GU451740] [GenBank:GU451733]

Oman2	Wadi Qab	22°50'13.7"; 59°14'34.7"	30/11/2004	[GenBank:GU451741] [GenBank:GU451734]

Philippines (Luzon)	Bulan	12°39'55.0";123°54'38.5"	12/03/2004	[GenBank:GU451748] [GenBank:GU451730]

Sri Lanka	Kekirawa	08°01'04.7"; 80°36'16.3"	10/07/2005	[GenBank:GU451742] [GenBank:GU451735]

Thailand	Khon Kaen	15°50'06.0";103°23'16.0"	08/03/2000	[GenBank:GU451743] [GenBank:GU451736]

Thailand	Phitsanulok	16°49'45.0";100°17'30.0"	14/06/2001	[GenBank:HM104223] [GenBank:HM104222]

*Radix auricularia rubiginosa*				

Thailand	Udon	17°10'19.0"; 102°26'25"	29/05/2001	[GenBank:GU451737] [GenBank:GU451749]

*Biomphalaria glabrata*				

Brazil	-	-	-	[GenBank:NC_005439]

All sequences are new data collected for this study, with the exception of the *Biomphalaria *sequence which was obtained from preexisting data in GenBank. GenBank accession numbers are given for all data.

Uncorrected *p *distances were obtained from PAUP* and transformed to percentage sequence divergence estimates. The use of this simple (there was no correction for transitional bias) distance measure was to afford comparison with other studies on inter and intraspecific variation among freshwater pond snails. The average divergence among ingroup taxa was 3.123% (range from 0.095 to 8.385%). The largest divergence was observed between the Bangladesh population and the other ingroup populations (excluding Assam), average 7.871% (range from 7.617 to 8.385%). This was followed by the divergences between the Assam population and the other ingroup populations (excluding Bangladesh), average 6.546% (range from 6.345 to 6.724%). In contrast, the divergence between the Assam and Bangladesh populations was only 4.16%. The divergence between *Biomphalaria glabrata *and *Radix a. rubiginosa *was 16.9%.

### Phylogeny reconstruction

#### Maximum likelihood (ML) method

**Figure 1:**
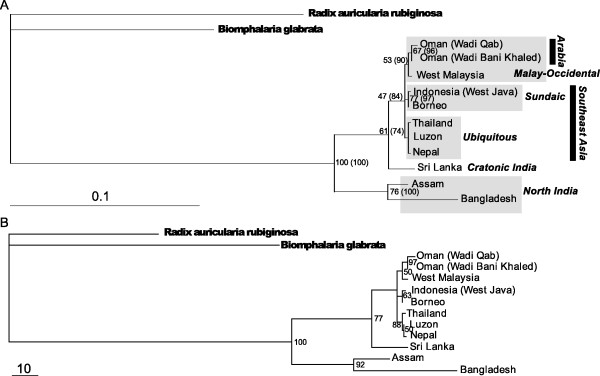
**Phylograms estimated for the *Indoplanorbis exustus *populations sampled (with clade support values) **A. Phylogeny estimated by maximum likelihood. Numbers assigned to each node are bootstrap support values in the maximum likelihood analysis (5000 replicates); the numbers following in parentheses represent the posterior probability that the hypothesis represented by this bi-partition, and under all parameters of the model, is correct, given the observed data in a corresponding Bayesian analysis implemented using MrBayes. Clades referred to in text (and Table 2) are given in italics and their bounds indicated by grey or black shaded boxes. B. Phylogeny estimated by a Bayesian method implemented in P4 and with a polytomy prior set to *e*^1.11^. Numbers assigned to each node are posterior probabilities for the corresponding bi-partition. The posterior probabilities are scaled here to range from one to 100.

A phylogeny was estimated for all taxa (with unique haplotypes) using PAUP*. An LRT comparing the TIM + G and TIM + ss models for the *cox*1 data indicated a significant difference between them (χ^2 ^= 336.627 *P *< 0.0001, d.f. = 2) favouring TIM + ss, consequently this model was used for the *cox*1 partition. Here ss refers to the estimation of codon position specific rates. An LRT of the molecular clock hypothesis failed to support the hypothesis that the different lineages had been evolving at the same rate (-ln likelihood with a clock enforced 3218.4030, without clock 3207.4237; χ^2 ^= 21.96, *P *= 0.0247). Figure 1A shows the phylogeny estimated by direct ML for these taxa. The most basal taxa are the northern India populations (Assam and Bangladesh), followed by South Indian (Sri Lanka). From this "Indian" root, a SEAM clade arises; this clade is not fully resolved and comprises a trichotomy formed by a Oman/Malaysia clade (Malay-Occidental, MO), a Sundaland (Sundaic) island clade (Indonesia and Borneo) and a "ubiquitous" clade (including Laos, both Thai populations, the Philippine population and Nepal). Here we define Sundaland as the islands, which rest on Asia's shallow continental shelf, namely the Malay Peninsula and maritime Sundaic islands of Sumatra, Java, Borneo, and the surrounding smaller islands. Aside from the placing of 2 taxa (Malaysia and Sri Lanka), this phylogeny is consistent with the *a priori *hypothesis for a radiation out of central Asia via India, and driven by solely geophysical factors. The occurrence of Nepal in the "ubiquitous" clade with Thai taxa was unexpected. We doubt that this was an error, as the Nepal samples were processed almost a year apart from the Thai and Philippine samples, also the sequences were not identical to any other taxon in that clade (and no other taxon showed intra-population variation).

#### Bayesian method with P4

Initial runs with a burnin of up to 2.3 million generations, up to 12 million sampled generations, and with TrN+I, F81, HKY, HKY + G and F81 models specified for codon positions 1, 2 and 3, *rrn*L and gaps, respectively, indicated a possible burnout problem (the likelihood reached a peak, then fell and then plateaued) with failure to reach true convergence. Burnout can be a sign that the Markov chain has been distracted from the ML region by regions of lower likelihood but higher posterior probability mass. The result of this can be apparent exclusion of unbiased tree lengths from the 99% credible interval, where this interval becomes so wide that the chain is unlikely to sample it evenly[36]. This problem is often caused by issues related to model specification, such as the use of overly complex models. To overcome problems with convergence and burnout the following changes were made. The chain temperature was lowered from 0.2 to 0.1 (thus making cold and heated chains more able to exchange states, thus increasing acceptance rates of proposals); the model was simplified by substitution of TrN (BIC 1050.244) for TrN + I (BIC 1057.008) (a move also possibly reducing Long-Tree (LT) error); and a user starting tree was specified (again potentially reducing LT error). The starting tree was based on the tree in file R1 (see Additional file 2) but with all non-Indian taxa (and Nepal) in one unresolved clade, branch lengths were otherwise taken from the PAUP ML tree. The starting tree was edited to have a non-bifurcating root.

After implementing the above moves apparent convergence was achieved with a burnin of 4 million generations. Posterior probabilities were then estimated over 10 million generations beyond the assumed point of stationarity. The final polytomy prior was set to 3.038 (varying the prior from e to e^2 ^had no significant effect on the phylogeny). The relative rates of the 3 codon positions were 0.253479, 0.283483 and 2.5462; the latter value was the highest rate of any partition and corresponded to the third codons, consequently there was no indication of LT error. The resulting phylogeny (see Figure 1B) was identical to that of the ML method except that the ubiquitous clade was resolved (Thai, (Luzon, Nepal)).

#### MrBayes

The final run conditions were: GTR, F81, HKY, HKY + G and F81 (for the *cox*1 codons and the *rrn*L partition, respectively); the chain temperature was 0.1; the covarion model was applied to the first 4 partitions (invoking + I) the BF comparing this move with the simpler model was 183.46 in favour of the covarion model; the ASPSD was 0.005028; the PSRF values ranged from 1.000 to 1.009 (mode 1.003); the burnin was 2.7 million generations, with 10 million generations sampled post burnin; a random starting tree was used. GTR was used for the first partition because of the limited range of models specifiable in MrBayes 3. The topology of the estimated phylogeny was identical to that of the ML method, with almost identical relative branch lengths.

#### Parsimony method

POY4 was used to implement a parsimony method using dynamic homology characters with direct optimization (i.e., no *a priori *multiple sequence alignment was required). In the initial sensitivity analyses the Navajo Rug indicated that no partition was overly sensitive to variations in weighting model over the range tested (see Additional file 3), and that, whilst the *cox*1 data appeared best for resolving more recent radiations, the *rrn*L data appeared best for recovering the deeper phylogenetic divisions. The maximum MRI values were obtained for schemes with gap cost = 1 and did not decline with Tv cost until costs exceeded 32. The maximum MRI for *cox*1 and *rrn*L was greater than that seen when the *cox*1 first and second codon positions were partitioned separately from the third positions (0.45 *vs *0.41; see Additional file 4). As the Tv cost weighting schemes 2, 4, 16, and 32 were equivalent in the MRI testing, the POY tree searches were repeated with gap cost = 1 and all 4 of the Tv weightings in turn. The *cox*1 and *rrn*L loci were input as two separate partitions. The *Radix *sequences were set as the outgroup. A single most parsimonious tree was found. The resulting cladogram was identical in topology to the phylogeny estimated by the ML method. The same tree was chosen either with or without the iterative pass search.

Clade posterior probabilities estimated using MrBayes were all high; bootstrap support values from the direct ML method were high (>70%) for all clades except for the Malay-Occidental (MO) clade (53-67%), the SEAM-Sundaic clade (47%) and the Sri Lanka-SEAM-Sundaic clade (61%). Posterior probabilities for clades recovered using P4 were also high except for MO (50%) and Luzon/Nepal (50%); this was with a polytomy prior set, with polytomies excluded (as in MrBayes) clade support increased (all above 70%) (see Figure 1). Jackknife support on the maximum parsimony tree showed a similar pattern to the Mr Bayes tree.

### Hypothesis testing

#### Shimodaira-Hasegawa test (SH-test) and topological method (TM) tests

A constraint tree was first drawn to describe a plausible hypothesis for the radiation of *Indoplanorbis *based only on palaeogeographical and climatological events (see Additional file 2). This tree assumes a central Asian origin for the genus, with colonization of India and then SEAM and finally the Sundaic islands. The events used to shape this tree were: 25 Ma Arabia-Africa separation (*Indoplanorbis *diverges from other bulinines)[37]; 20 Ma orginally Indian taxa enter Sundaland via extended Brahmaputra-Irrawaddy and become isolated there[38]; 3 Ma taxa isolated on Borneo/Indonesia as these terrains separate from Malaysia[39]; <0.5 Ma SEAM taxa isolated at end of Mid-Pleistocene Transition (MPT)[40]. The end of the Arabian humid interval (3.5 - 1.2 Ma[37]) was used to date the Oman radiation. The branch lengths of these trees were then rescaled to within the range of those found in the ML tree from the PAUP* analysis. The Nepal taxon was moved from the Indian clade to the Sundaland clade to follow the indications of the (unconstrained) ML tree search.

The log likelihood for the geophysical hypothesis (tree) was -3218.32 *P *= 0.358, indicating that it is an equally likely explanation of the data as the unconstrained tree (all other "best" trees were significantly worse). In TM test (using the same hypothesis tree) the posterior probability of the geophysical hypothesis was 533/47779 trees, i.e., *P *= 0.0112. These results suggested that the true phylogeography might not differ significantly from the *a priori *hypothesis, but that trees of exactly that topology were not well represented in the posterior (i.e., the phylogeography indicated by the data is significantly different from that of the central Asia-Brahmaputra-SEAM hypothesis).

#### Estimation of divergence times with BEAST

For these estimates all 15 taxa were included but gaps were excluded (as missing data). Initial runs with a Yule tree prior and an uncorrelated log-normal (relaxed) clock model showed that the ucld.stdev was >1.0 and its confidence interval did not come close to zero (mean 2.84816 HPSD ranged from 1.80335 to 3.7994); this suggested that the data were not sufficiently clock like for analyses using a strict molecular clock. A 2 partition model (by locus) with TIM + G and HKY + G was compared with a by codon model (TrN, F81, HKY, HKY + G) the latter appeared preferable (BF 87.88). Other moves, such as TrN+I or HKY, HKY did not result in significant improvement. Consequently, TrN, F81, HKY, HKY + G was used in the main analyses. The prior on the mean rate was a normal prior with mean equal to the average of published *rrn*L (one rate) and *cox*1 (2 rates) clock rates for freshwater snails and S.D. Set such that the 95% confidence interval spanned twice the range of these 3 values. The 3 rates were as follows (in substitutions per site per year × 10^-8^): 1.097 for *rrn*L, triculine snails (Gastropda: Pomatiopsidae) over the early Pleistocene[41]; 1.83 for early Pliocene Hydrobiidae[42]; and 1.62 also for Hydrobiidae[43]. These rates were estimated following the simple point estimation method of Edwards and Beerli[44], but are useful as a prior in this study. The other initial empirical prior was one on the height of the tree; this was a 100 to 350 Ma uniform prior. A more informative normal prior of 200 Ma S.D. 30 Ma was also used, but there was no marked difference on the estimation of mean rate or other TMRCAs (Time to Most Recent Common Ancestor). The informative prior was based on a Triassic/Jurassic boundary date for the divergence of the Planorbidae and Lymnaeidae[24]. Runs were also performed using an NPRS-transformation of the ML tree from PAUP as a starting tree (scaled to a height of 250 Ma), but, as this had little effect on run performance, the final runs used random starting trees. Runs using only a prior on tree height or on mean rate (but not both) gave implausible results. Sampling from the prior only (with conditions otherwise set to those of the final runs) also resulted in implausible results and TMRCA estimates very different from those obtained after observing the data. Each run was repeated with 3 different starting random number seeds the results of each run were compared and then combined for parameter estimation from the posterior distribution.

The final runs used a coalescent model, with the prior assumption of constant population size (throughout the genealogy), for the starting-tree prior and an uncorrelated log normal relaxed clock model. TMRCAs were estimated for the most basal divergences first, with a prior on the TMRCA of the outgroup as described above. The outgroup was then removed and the more recent divergences were estimated with a prior on the TMRCA of the Sri Lanka population and all of the SEAM-Sundaic populations (this was the split subsequent to the outgroup); this prior was the estimated value from the first runs (with all 15 taxa). The outgroup was removed because its inclusion results in undesirable time depth of the phylogeny; this led to underestimation of the overall mean rate and inflated time estimates for the more recent divergences among the ingroup. The McMC was 10 million generations each run (3 runs were combined), with a burnin of 10%. All ESS values were >500. The posterior distributions were examined using Tracer[45] and no distribution was seen to be cut off by its prior or show signs of failure to converge (rising likelihood). The estimated TMRCA values are given in Table 2. TMRCA estimates for an exponential distribution clock model were also examined to ensure they agreed with those in Table 2; this was because BF comparisons of the two models indicated they were equally fitting (BF 0.16 in favour of the log normal model. It was found that simpler tree priors (Yule and Birth-Death models) gave implausible TMRCAs, with coalescent priors performing better. The wide range of the HPDs in for the divergence time estimates in Table 2 reflects the uncertainty inherent in all molecular date estimates; this is not unusual and is a realistic feature of this method of analysis.

**Table 2:** Results of a Bayesian estimation of divergence times

Parameter	**Mean **± **S.E**.	ESS	95% HPD Lower/Upper
-ln(likelihood)	2018.99 ± 0.55	3517.56	2028.77/2007.54

mean substitution rate	1.37 ± 0.01	4896.57	0.02/2.43

tmrca(Outgroup)	186.99 ± 0.03	17814.11	180.58/194.00

tmrca(Ingroup)	6.93 ± 0.42	1085.77	1.09/20.42

tmrca(North India)	2.84 ± 0.13	2944.97	0.01/8.83

tmrca(Cratonic India)	2.48 ± 0.09	2796.45	0.13/9.48

tmrca(Southeast Asia)	0.96 ± 0.04	2614.02	0.07/3.37

tmrca(Malay-Occidental)	0.45 ± 0.02	3219.78	0.01/1.5

tmrca(Arabia)	0.18 ± 0.01	4481.45	0.00/0.60

tmrca(Sundaic)	0.17 ± 0.01	4426.12	0.07/0.57

Tmrca (Ubiquitous)	0.16 ± 0.01	3697.91	0.01/0.52

Time estimates are given in millions of years (Ma) for nodes representing the most recent common ancestor of relevant clades. The mean substitution rate is expressed per 100 bases per Ma. ESS, effective sample size (corrected for auto-correlation); HPD, the 9% highest posterior probability density (equivalent to a confidence interval); SE, standard error of the mean; TMRCA, time to most recent common ancestor of the taxa in the clade. Here Southeast Asia refers to SEAM (Southeast Asian mainland) and Sundaic.

## Discussion

### Phylogenetics

The topological-method hypothesis test rejected the *a priori *(central Asia-Brahmaputra-SEAM) model; this suggested that the phylogeography of these snails differed significantly from the history of other snails such as the Triculinae[46], which are also found in India and the SEAM. The phylogenies estimated for *Indoplanorbis exustus *offer strong and concerted support for an origin of the South and Southeast Asian radiation in Northern India; the Assamese and Bangladeshi populations form a robust basal clade. The cratonic Indian (or at least South Indian/Sri Lankan) populations radiated later. Sri Lanka was intermittently linked to mainland India by extensive land bridges until about 165 Ka[47], consequently the Sri Lankan population is considered as phylogeographically South Indian. The most recent radiation could only be partially resolved by the present data, resulting in a trichotomy of Malay-Occidental (MO), Sundaic and ubiquitous clades. The ubiquitous clade had representatives on Thailand and Laos (SEAM), Luzon (Philippines) and Nepal (Himalayan frontal ranges). The colonization of Arabia (and probably also Africa) appears to be most recent and this agrees with earlier assumptions based on biogeography only[17,18,48]. The suggestion of an Indian origin also agrees with earlier biogeographical hypotheses[48]. The derivation of the Luzon/Nepal clade from the SEAM element of the ubiquitous clade was less well supported; this clade was only supported by the P4 analysis; however, it is reasonable to accept that the extralimital taxa originated on the SEAM.

The relative depths of divergences in the phylogeny suggest that *Indoplanorbis *comprises a single species, save for possibly the northern India lineage, which forms a cohesive basal clade. If the Assam/Bangladesh taxon is a distinct species this may explain the apparent stasis in the northern Indian radiation for a relatively long period, followed by a more recent and sudden radiation of *Indoplanorbis *outside India. Also worthy of note in this context is the observation that infection prevalences in natural populations (with *Schistosoma spindale*) were always >8% in samples (of over 200) of snails collected in Thailand and Laos (this study), but are reportedly much lower in *Indoplanorbis *sampled in Assam (2.2% [8]) and prevalences of under 1% seem typical in India[4]. In addition, the Assam/Bangladesh taxa exist in a region of current (and historically) higher rainfall than other parts of India (except for the Ghats) and Southeast Asia where the samples were taken (see Background, first section). Although the concept of critical levels of sequence divergence (genetic distances) that can be used to delimit species boundaries is generally inappropriate, such data can be used to differentiate species in combination with other kinds of observations. Typical interspecific divergences of 1.9-6.9% have been reported for *Biomphalaria*[49] (for the mt 16S and nuclear ITS loci) and, as these snails have similar ecologies to *I. exustus*, these values could be used as indicators in the present study. Genetic distances for the related snail *Bulinus *have been reported for the *cox*1 locus. The mean distance within *B. forskalii *from different countries was 4.2% and for *B. senegalensis *1.7%. The divergence between two closely related *Bulinus *species, *B. camerunensis *and *B. forskalii*, was 3.96%[50]. In view of these reports, and considering the differences between those and the present study, a divergence greater than 6.5% might be considered suggestive of polyphyly in the present data. In the present study only the Assam and Bangladesh taxa showed such high divergences from other ingroup taxa. Such observations, taken together, may be sufficient to warrant a detailed morphological comparison of the Assamese (and cratonic India) and SEAM taxa in order to confirm their conspecific status. In the present work morphological study was restricted to only that sufficient to be sure that the snails collected were no other known species aside from *I. exustus*.

**Figure 2:**
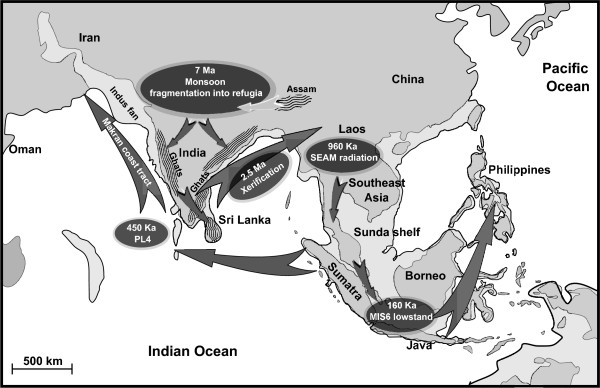
**Semi-schematic summarizing a phylogeography for *Indoplanorbis***. The radiation is assumed to have originated in the Assam region of Northeast India and initial divergence to have been triggered by the onset of the Asian monsoon and a change from rain forest to drier savanna. More humid refugia are shaded with wavy lines. Arrows show subsequent dispersal tracts. Pleistocene land area is shown by lighter shading extending from the modern coast lines. Pleistocene coastlines are indicated by solid lines, modern coast lines by broken lines. International boundaries, scale and geographical features approximate.

The occurrence of the Nepal sample in the SEAM clade, and Sri Lanka in the SEAM-Sundaic clade is similar to other disjunct distributions reported for a variety of early Pleistocene organisms, where certain Indian taxa show greater affinity with Southeast Asian taxa than with neighboring Indian forms. The Asian langurs[51], certain bird species[52] and *Macaca spp.*[53] show such disjunct distributions. The phylogeny of the snail *Paracrostoma *Cossmann, 1900 (Caenogastropoda: Pachychilidae) and other pachychilids shows a similar disjunction between Indian and the SEAM[54]. Although these authors advanced a slightly different phylogeographical hypothesis for this taxon, *Paracrostoma *showed similar disjunct India-Southeast Asia affinities in their study as observed for *I. exustus *in the present study . These disjunct distributions are proposed to have arisen following Plio-Pleistocene (5.1-1.6 Ma) aridification of southern India with a shift in the predominant vegetation cover type from tropical forest to Savannah, with "wet-zone" species becoming confined to and migrating along refugia of tropical forest in the Western and Eastern Ghats and Sri Lanka[52] (Figure 2). Wet-zone is defined as regions with annual precipitation of >250 cm and dry-zone as areas with 50-100 cm or less, the SEAM is wet-zone. This "dry-zone refugia" hypothesis would allow migration of (wet-zone) taxa from Southeast Asia, via Myanmar and into Sri Lanka and mainland India. Such migration would be facilitated by Quaternary glacial eustasy, as lowered sea levels exposed continental shelf areas providing terranes of low relief and suitable dispersal corridors. For snails radiating in the early Pleistocene such a disjunct distribution would be correlated with fluctuations in sea level during glaciations around MIS22 (Marine Isotope Stage 22)[55].

### Molecular dating

The results of molecular dating using the present data and a Bayesian approach with a relaxed clock model are given in Table 2. The mean substitution rate of 1.37 × 10^-8 ^substitutions/site/million years agrees well with published estimates for freshwater snails at these loci[43]. The divergence of the ingroup is dated at around 7 Ma. This date corresponds with accelerated Himalayan uplift (at 8 Ma) and the associated establishment of a monsoon climate in Asia[56]. Consquently a major climatic change occurred in northern India at this time, with the appearance and dominance of C_4 _plants in the flora and associated deforestation[57]. *Indoplanorbis *requires humid tropical environments and Indian populations could have been fragmented into refugia at this time. Assam is regarded as a distinct from the drier Sikkim Himalaya (which includes Nepal)[58]. In view of this it is plausible that the initiation of a monsoon dominated climate triggered the divergence of the Assam/Bangladesh (Table 2, North India) and the cratonic Indian and SEAM-Sundaland clades at 7 Ma. The dating suggests that proto-*Indoplanorbis *remained unchanged on the Indian craton for almost 100 Ma; thus the divergence at 7 Ma may have been a result of some adaptation to more arid conditions, such as the appearance of a new ecological race (if not species) adapted to drier conditions. As mentioned above, the Assam region, and the part of Bangladesh where the samples were taken, has a year round more humid climate than the areas of SEAM that experience an annual dry season. The divergence of the Assam and Bangladesh populations after 2.84 Ma could be attributed to mid-Pleistocene changes in river courses associated with rapid uplift of the frontal range of the Himalaya[59] and uplift of the Silong Plateau and parts of the Meghalaya Plateau and Khasi hills which separate these regions today[60].

The next major divergence event is the divergence of the cratonic India clade (Sri Lanka) from the Sundaland clades; this is dated at around 2.5 Ma. It is known that significant Asian monsoon intensification occurred prior to the major glaciation at around 2.7 Ma; this triggered an extremely arid 1.4 Ma interval[61]. This dry period is likely to have forced Southern Indian and SEAM populations further into dry-zone refugia, thereby promoting additional divergence. The end of the arid period may then have led to the explosive radiation of the SEAM and Sundaic taxa at 960 Ka. Note that by 900 Ka sea levels were again quite high[62] and this would prevent back migration after range expansion (thus further facilitating divergence). The next dated event is the divergence of the MO clade at 450 Ka, with the Arabian populations diverging from a Sundaic (Malaysian) ancestral form. This interval (PL4) is associated with very low sea levels[63], milder interglacials (with more extensive wetlands) after 430 Ka, and maximum vegetation cover in Arabia around 500 Ka (MIS13)[64]. These events could facilitate migration of Sundaic taxa, from the western rim of the Sunda shelf (Sumatra), across exposed regions of the Indian Ocean, then around the exposed Indus fan and into Arabia or Africa (Figure 2). A Makran coast tract, however, raises the question as to why the Sundaic lineage did not establish in southern India or Pakistan/Iran, as it would have passed through these regions. The absence from southern India may be explained by ecological adaptation of the Sundaic form to SEAM habitats. The fact that the migration would have been across low lying wetland regions that were later below sea level may explain the absence from Pakistan/Iran. When these terranes were next exposed winter conditions were much colder than prior to the Mid-Pleistocene-Transition (MPT)[64]. In addition, after 500 Ka sea levels were never as low for such sustained intervals[65]. The MO clade is then dated as further diverging at 180 Ka into two clades in Oman, which were represented by two populations in different wadi systems, separated by 30 km of extremely arid mountains. The date 180 Ka coincides with the end of the longest Pleistocene humid period in southern Arabia (200-180 Ka)[66]. It is possible that the return to much drier conditions and contraction of vegetation cover blocked further immigration of snails to the region and thereby isolated the coastal (Wadi Qab) population from the more interior (Wadi Bani Khaled) population. Humid periods also appear to have occurred at 160-130 Ka, 82-78 Ka and [67]10-6 Ka[68], but these were a series of shorter lived wet periods. The mountains between the two Oman populations suggest that the two sites were colonized via different tracts, that probably originated from the same source in Makran Iran (across the exposed Oman and Persian gulfs), one passing inland and one coastal.

The most recent radiations appear to be the Sundaic additions to the range; these being almost isochronous at around 165 Ka. This interval corresponds to the MIS6 glacial at 195-130 Ka during which sea levels reached minima of around 120 m below present levels (*c.a. *140 Ka)[65]. At such times of eustatic minima the Sunda shelf between the SEAM and Java and Borneo was exposed (see Figure 2) and island arcs (volcanic) provided land bridges towards the Philippines, such that rafting and transport by birds could bridge the shorter stretches of sea separating these islands. The presence of the Nepal taxon in the SEAM clade rather than the Indian clade could be explained by glaciation, and or hyper-aridity eliminating snail populations in Nepal. There is some evidence for late Pleistocene glaciation in southern Nepal, but the Brahmaputra valley and areas South of the Nepal: Assam border appear to have remained unglaciated[69]. There is also evidence for a hyper-arid climate in West India 18-13 Ka, but Assam appears to have provided a more humid refugium[70]. If snail populations became extinct in Nepal they could have been replenished by colonization from the SEAM by populations which were not sampled in the present study (e.g., from Myanmar). As mentioned above the SEAM lineage contains the most widely dispersed taxa and this suggest some adaptation in this clade which makes it more invasive and possibly able to out compete Indian clade taxa.

## Conclusions

The findings of the study suggest an Indian origin for the *Indoplanorbis *radiation at the Miocene-Pliocene boundary. The radiation appears to have been triggered by the switch of the Asian climate from a planetary to a monsoonal climate. The antecedent taxa were probably adapted to life in humid and partially forested tropical to sub-tropical environments, and now appear restricted to refugia in Assam and Bangladesh. Further intensification of the Asian monsoon at 2.5 Ma appears to have triggered divergence of a peninsular Indian taxon; this taxon may have been better adapted to the savanna habitats that replaced rain forest as the dominant vegetation type in cratonic India at that time. *Indoplanorbis *then appears to have entered the Southeast Asian Mainland (SEAM) by 1 Ma. The end of a very arid period then triggered divergence of the snail on the SEAM at 960 Ka, but the SEAM lineage may already have acquired additional adaptations to aridity which allowed it to colonize new areas more effectively and to live in regions with pronounced dry and wet seasons more readily than the original Indian taxa. The SEAM lineage first colonized Arabia (possibly via Sumatra, island chains and the exposed Indus fan and Oman gulf during periods of low sea level) at around 500 Ka and then the Sundaic islands. Severe tradewinds in MIS6 could have facilitated this westward migration[71]. It is possible that there have been multiple colonization events, out of the SEAM, over the Pleistocene glacial cycles, with replacement of ancestral forms by ever better adapted (to dry conditions) forms; such loss of ancestral polymorphism could make the colonization of Arabia from the SEAM appear to predate that of the Sundaic islands, when the Sundaic islands may have been colonized first. The phylogeography hypothesized here is not proposed as a definitive explanation, but only to demonstrate that the major relationships, tracts of dispersal and timescale are plausible. No doubt alternative palaeogeographical and climatic events could be put forward to explain the same phylogeny.

The low eustatic sea levels of the Würm glacial maximum allowed human migration from central Asia, across northern India and into Myanmar and the SEAM via Assam (130-70 ka). Inter pluvial low sea levels could have permitted colonization of Indonesia and Borneo, even as far south as Australia[72]. Recent studies on the evolution of human mtDNA haplogroups suggests that Sundaland was colonized by humans (possibly from northeast SEAM) over 50 Ka[73]. Humans did not develop a significant maritime technology until 60 Ka[74]; this would have made the colonization of the Philippines possible. Although such human migrations are recorded for the middle palaeolithic, the domestication of livestock, and their subsequent trade, did not begin until around 10 Ka[75]. Consequently, an anthropogenic radiation of *Indoplanorbis *would be expected to accelerate and diversify mostly in the last 10 Ka and a human-mediated dispersal would be expected to show a phylogeny with less phylogenetic depth than one driven by geophysical events.

The timescale of the radiation, which is estimated to be Pliocene to late Pleistocene, entirely predates the radiation of the genus *Homo *in Asia and the human maritime culture. Consequently, the results of this study support geophysical and climatic forces, rather than human activity, as drivers of the dispersal of *Indoplanorbis *across Asia and its arrival in Arabia. In contrast, the results could not distinguish an Indian rafting hypothesis from an Africa to India migration via central Asia (because the phylogeny shows a complex migration out of India, rather than an informative tract such as Assam-SEAM-Sri Lanka). Interestingly, the radiation of *Indoplanorbis *does not appear to track that of *Schistosoma spindale*, which has been dated at 0.25 Ma for the divergence of Bangladesh, Thai and Sri Lankan populations[29] this suggests that the host-parasite association here is not tightly co-evolved. As indicated by the phylogeny, *Indoplanorbis exustus *appears to comprise two or three ecological races or ecotypes. These races show different habitat requirements (the SEAM lineage appears to have a wider ecological tolerance) and some parasitological differences (the SEAM lineage shows higher prevalences with *S. spindale*). The high levels of sequence divergence between the Northeast India populations and the other ingroup taxa also suggest that these taxa may be a different species from *I. exusutus*. Consequently, there is a case for a detailed morphological study to confirm that *Indoplanorbis *is truly monospecific. This is in addition to the need for additional sampling of *Indoplanorbis *from Afghanistan, Myanmar and western India - to distinguish an Indian rafting hypothesis from a central Asian tract from Africa, to test theories on the origin of the Nepal population, and to confirm that the inclusion of only a Sri Lankan population correctly represents the cratonic Indian lineage. Under the proposed phylogeography it is conceivable that southern peninsular India could have been colonized by snails with SEAM clade adaptations distinct from that of the wetter more forested Sri Lanka.

## Methods

### Sampling

Samples were taken in 10 countries so as to cover most of the range of *Indoplanorbis exustus *(see Table 1 for details of taxa and sampling). No samples were taken in Africa, as these populations were assumed to be represented by the samples from Arabia. Identification of taxa followed published accounts[17]. Whole snails were fixed in 10% ethanol directly in the field. A small subsample of each population was taken in 1% neutral formalin to aid identification. DNA was extracted from individual snails by a standard method[76]. Sequence variation was assessed at two loci, being partial sequences of the mitochondrial (mt) cytochrome oxidase subunit I gene (*cox*1) and the mt large ribosomal-RNA gene (*rrn*L or 16S). Sequences of the oligonucleotide primers used in the PCR for the amplification of these loci are published elsewhere, for the *cox*1 amplifications the primers HCO-2198 and LCO-1490 were used[77]. For the *rrn*L locus the primers of Palumbi *et al. *(1991) were used[78]. 2 to 8 snails were sequenced for each locus/population (mode 4 *rrn*L and 2 *cox*1). Mitochondrial loci were chosen because with their maternal pattern of inheritance and therefore smaller effective population size, these loci may be expected to evolve more rapidly and be better suited to estimation of intraspecific phylogenies and more recent (i.e., post-Miocene radiations). In addition, these loci have proven useful in such studies of gastropods and molecular clock rate data are available[24,42,43,46,79,80]. Total genomic DNA was used as a template for PCR amplification on a Progene thermal cycler (MWG) employing standard PCR conditions[81]. Unincorporated primers and nucleotides were removed from PCR products using the QIAQuick PCR purification kit (QIAGEN). Sequences were determined bidirectionally, directly from the products by thermal- cycle-sequencing using Big Dye fluorescent dye terminators and an ABI 377 automated sequencer (Perkin-Elmer), following procedures recommended by the manufacturers. Sequences were assembled and aligned using Sequencher (version 3.1 Gene Codes Corp. Ann Arbor, Michigan). DNA sequences for both strands were aligned and compared to verify accuracy. Controls without DNA template were included in all PCR runs to exclude any cross-over contamination.

A multiple sequence alignment was generated for all taxa for each locus and the *cox*1 and *rrn*L sequences were then concatenated to form a combined data set. No intrapopulation variation was found. Corresponding sequences were obtained for *Radix auricularia rubiginosa *(Michelin, 1831) (Lymnaeoidea: Lymnaeidae) in this laboratory, as outgroup sequences. Additional outgroup data were obtained for *Biomphalaria glabrata *(Say, 1818) (Planorbidae: Planorbinae) from the GenBank [GenBank:NC_005439][82].

### Preparation of data

As an initial test for phylogenetic signal in the data, a Permutation Tail Probability test (PTP)[83,84] with 50000 replicates (each of 100 random additions), was performed on the ingroup. Although often criticised for being too liberal[85], the PTP-test is useful as a first step in phylogenetic analysis[86], as rejection of the null hypothesis here suggests that the real data may be better than randomly permuted (phylogenetically uninformative) data. In addition Tajima's test for neutrality (based on the total number of mutations) was performed using DNAsp (v. 5.10.01)[87]. The data were tested for substitution saturation using plots of the numbers of transitions (Ts) and transversions (Tv) against the maximum likelihood (ML) genetic distance[88]. The indications of these plots were further evaluated using the entropy-based test[35] as found in the DAMBE (v. 4.5.20) software package[89], which provides a statistical test for saturation. Aside from the parsimony analyses, gaps were coded as a binary matrix, and all characters were run unordered and equally weighted. Indels for the sequence alignment appeared to be homologous (they were short and of equal length in all taxa) and alignment errors were unlikely (the sequences aligned readily in Sequencer under stringent parameters). In addition, recurrent mutation is less likely to significantly affect the analysis of intraspecific phylogenies and there was no reliably tested approach for choosing a particular model. Consequently, gaps were coded simply in a binary, presence/absence, manner rather than using SIC or other more complex coding methods[90].

### Choice of substitution model and data partitioning scheme

The incongruence length-difference (ILD) test[91] as implemented in PAUP* (v. 4.0b10; [92]), was used to test for homogeneity between the *cox*1 and *rrn*L data partitions; the test was applied to informative sites only[93]. A suitable substitution model was selected for each data partition using an hierarchical test of alternative models as implemented in Modeltest (v. 3.7)[94] and MrModeltest (v. 2.3.)[95]. The two applications use different sets of hierarchies for the comparison of models[96], thus the additional use of MrModeltest not only provided models which could be implemeted in MrBayes, but also could highlight cases where Modeltest might fail to select a class of models due to the hierarchy of comparisons. Models were selected first by BIC, then by second order AIC, then (in the case of apparent equivalence of two models) by the number of free parameters (although AIC may also penalize extraneous parameters). There was no disagreement between Modeltest and MrModeltest in cases where a model considered by both applications was chosen by Modeltest. Molecular evolution of indels was modeled by an F81 model. For the calculation of the BIC, the sample size was set as the number of characters in the alignment.

### Phylogeny reconstruction: parameters and priors

Phylogenetic estimation used three different approaches; this strategy was adopted to look for resilience of the hypothesis to changes in method and associated assumptions. The rationale was that any clade that was represented in phylogenies found by all methods (and well supported) would be a reliable reconstruction of phylogenetic history for these taxa. The use of a direct ML method and Bayesian methods also allowed comparison of estimated branch lengths, as Bayesian methods (as implemented in MrBayes) have been known to erroneously converge on LT solutions in cases where partitioned data are described by complex models with many parameters close to nonidentifiability[36,97]. The ILD test comparing the *cox*1 and *rrn*L loci was significant (*P *= 0.02), so the two partitions were modeled separately. The starting models indicated for the ML analyses were as follows:

All sites: TIM + G

*cox*1 first codon positions: TrN + I

*cox*1 second codon positions: F81

*cox*1 third codon positions: HKY

*rrn*L: HKY + G

Indels: F81

The F81 model for gaps was not chosen by goodness of fit tests, but it is the only appropriate model suitable for indels. Also, it is a monotone model fitting the similarly monotone substitution of a binary character evolving along a phylogeny[98]).

The following details the methods particular to each phylogenetic method. Analyses were time consuming and performed on several multiprocessor machines (4 × 2.44 GHz PCs), with output files pooled from multiple simultaneous runs where appropriate.

#### ML with heuristic search

Heuristic searches were performed (under the respective model and starting parameters indicated by Modeltest) using PAUP* initially with 1000 replicates, random addition of sequences (10 replicates) and tree-bisection-reconnection branch-swapping (TBR) options in effect. The TIM+G model was used, gaps were treated as missing data. The outgroup was forced to be monophyletic. The initial search rapidly converged on a single tree which was hit 100% of the time. Consequently, the search was repeated with nchuck = 10 and chuckscore = 3210 in an attempt to improve exploration of parameter space and find trees of better score. This was repeated with the monophyly constraint both on and off. A final search of 5000 replicates was then performed to increase the chance that the ML tree had been found. A likelihood ratio test for a strict molecular clock hypothesis was also made possible by repeating the final run with a clock constraint. Nodal support was assessed by bootstrap with 5000 replicates.

#### Bayesian method (BM) with McMC applied by P4

Bayesian phylogenetic methods are becoming increasingly popular in molecular systematics with the belief that they are not only faster in terms of computing time (for analyses with an equivalent level of confidence) but are also statistically superior to a solely ML method[99]. For example, such methods do not assume approximate normality or large sample sizes as would general ML methods[100]. BMs differ from direct ML methods in that the former consider the posterior probability of the model (with parameters) and tree after observing the data. The posterior probability of a hypothesis is proportional to the product of its prior probability and the probability of observing the dataset given the hypothesis (i.e., the likelihood of the hypothesis). Consequently, unlike direct ML, a BM allows incorporation of prior information about the phylogenetic process into the analysis. P4 (v. 0.87) was used as the primary method to apply the BM; this employs the same method as MrBayes[101] but allows consideration of unresolved trees (i.e. polytomies) and can implement all commonly used substitution models as well as any custom model the user might need to specify[102].

The priors specified for the BM generally followed the default values found in MrBayes; a flat Dirichlet distribution was set as the prior for the state frequency and for the rate set priors (*e.g*., revmat, tratio), an exponential prior was set on the branch lengths. A polytomy proposal was set as either zero (*i.e*., no favouring of multifurcations) or as *e, e*^1.11 ^*e2 *to examine the effect this has on the posterior probabilities of the clades found; this implements a move (proposed by Lewis et al., 2005) to counter the problem of the spuriously high posterior clade probabilities returned by MrBayes relative to corresponding ML analyses[103]. Model parameters and relative rates were set to be freely variable and unlinked; there were four discrete rate categories for the Г-distribution. Outgroup monophyly was enforced. The McMC was tuned to give proposal acceptance rates between 10 and 70% for each data partition.

Four simultaneous Markov chains were run (one cold, three heated) and trees were sampled every 10 generations, two such runs were performed simultaneously. Convergence of the McMC was assessed by plotting split support (at the Indonesia/Borneo node) for consensus trees over different generation time windows; the generation of convergence was considered to be that at which the support reached a plateau. In addition, a plot of the cumulative split frequencies from two simultaneous runs (which should cluster along the line y = x when both runs are converging on the same region of parameter space) was also used to determine the point of stationarity (see literature for details of rationale[104]). Generations prior to the inferred point of convergence (i.e., prior to stationarity) were discarded as a burnin.

#### Bayesian method with McMC applied using MrBayes

To afford comparison with more standard analyses and to confirm the findings with P4, the BM was reapplied using the popular package MrBayes (v. 3.1.2). Run conditions followed those of the P4 analyses, where possible. Unlike P4, the polytomy proposal for MrBayes is effectively fixed at zero and model specification was more limited than in P4. In order to help assess if runs had converged the average standard deviation of the split frequencies (ASDSF) was used to gauge the similarity of trees sampled by 2 independent runs and the Potential Scale Reduction Factor (PSRF) was used to compare the final mean parameter estimates (for the 2 runs), including the posterior probabilities of nodes[105,101]. The McMC was considered complete if this ASDSF was <0.01 otherwise the run (and burnin) was lengthened until the ASDSF was adequate. PSRF values close to 1.000 were also considered as indicating that convergence might have been achieved.

As noted above, it is possible for MrBayes (and P4) to converge on an incorrect LT solution where partitioned data are used with complex models. This can occur because the default starting tree used by MrBayes often has relatively long branch lengths for most data sets (this can draw the chain towards long-trees in the early stages of the run, such that convergence on the correct solution is never achieved). This problem may not be detected by either the ASDSF or PSRF[36]. To reduce the risk of this source of systematic error the following precautions were taken. Branch lengths of the final trees were compared with and without a user specified starting tree (the ML tree from PAUP was used for this). The relative rates for the partitions were examined because as the chain is drawn to LT solutions the relative rate of the partition with fewest variable sites is often inflated (and alpha falls to accommodate this). In the present study the expectation was that third codon positions should have the highest rates and *rrn*L among the lowest - deviations from this would be considered symptomatic of an LT error. Although the mean branch length prior in MrBayes was left at the default 0.1, that in P4 was lowered to 0.001 and the branch lengths of the final trees compared (following published suggestions[106]). There was an exponential prior on the branch lengths in MrBayes. In P4 Pinvar was also removed from the model for *Cox*1 first codon positions; this move was to reduce or detect any parameter correlation affecting convergence[107].

#### Parsimony method using POY

To provide an analysis free of the assumptions underlying ML methods, and any effects particular to the alignment inferred by Sequencher, the topology for the phylogeny of the snail populations was also estimated using a maximum parsimony approach implemented by POY (v. 4.1.1). POY was used to effect a direct optimization criterion, which performs simultaneous tree searching and sequence alignment. Direct optimization finds a set of parsimonious homologies for a set of sequences of different length by treating the full homologous nucleotide string as a character and each sequence as a state (i.e., the data are treated as dynamic homology characters)[108]. The data are partitioned in the sense that the homologies are not moved between them, but are combined during optimization in that each has the potential to influence the "alignment" of any other partition (strictly alignment is not performed, only implied). The data partitioning scheme and Tv/Ts weighting scheme and gap cost were determined by a MRI[109] and "Navajo rug" approach (a graphic plot where the parameter space is represented as a grid)[110]. The MRI provides only a rough measure of congruence among data partitions; however, the approach has performed adequately in empirical tests[111]. The Tv:Ts weightings considered were 1, 2, 4, 8, 16 and 32, and the gap costs similarly ranged from 1 to 64 (a total of 42 weight matrices). The gap opening cost was set to one. One Navajo rug was produced for each data partition (*cox*1, *cox*1 first and second codon positions, *cox*1 third codon positions, *rrn*L and *cox*1 + *rrn*L) and weighting scheme was plotted against clade, with each clade sored as monophyletic, paraphyletic or polyphyletic. Clades were taken from the ML tree found in the PAUP analyses as well as additional clades found on trees in the most frequent 5% of trees from the posterior distribution in MrBayes. The Navajo rugs were used to detect any unusual sensitivity of a particular data partition to changes in character weighting. The MRI was used to guide the choice of data partitioning strategy and weighting scheme.

The search strategy was as follows. Initially 250 random-addition (Wagner) trees were built. Alternate sub-tree pruning and TBR rounds of branch-swapping were then performed on all 250 trees until a local optimum was achieved (using default options in POY 4). In addition to the most optimal trees, all the suboptimal trees found within 5% of the best cost were also considered. Then all but the best 100 of all optimal and topologically unique trees were excluded from the analysis. The remaining trees were then subjected to 15 rounds of ratcheting, where 20% of the characters were re-weighted by a factor of 2. During ratcheting, the dynamic homology characters were transformed into static homology characters, so that the fraction of nucleotides (rather than of sequence fragments) could be re-weighted. 200 swappings of subtrees identical in terminal composition but different in topology, were then performed between pairs of best trees from the ratcheting step . These two steps were intended to increase the efficiency of the search of tree space. Each resulting tree was then refined further by local branch swapping under the default parameters in POY for swapping . The optimal and topologically unique trees were then reported. The resulting tree was then used as a start point for 2 rounds of optimization using iterative pass; this constitutes an extremely effective means of determining parsimonious cladogram costs[112]. Clade support was estimated by jackknifing of static transformed characters (5000 replicates, 50% of characters deleted).

### Hypothesis testing

#### Topological method

The posterior probability of a particular phylogeographical hypothesis was tested by determining the proportion of trees with this topology in the posterior probability distribution output from a tree search using P4. This was done by writing a constraint tree describing the hypothesis of interest and reading this into PAUP*.

#### SH-test [113]

Again a constraint tree describing the hypothesis of interest was written and used in the SH-test as implemented in PAUP* with full optimization, branch lengths used in likelihood calculation, and 1000 bootstrap replicates. The test compares between and among topologies from constrained and unconstrained searches; the set of "best" trees (from the 5% confidence interval of unique trees sampled from the posterior of the P4 search) and the ML tree (unconstrained) were used as the set of trees for this comparison.

#### Relaxed molecular clock methods (BEAST)

The program BEAST (v. 1.5.3)[114,115] was used to estimate the molecular clock rates and divergence dates (TMRCA of clades). BEAST implements a Bayesian method for the simultaneous estimation of divergence times, tree topology and clock rates; this method is currently considered superior to other approaches (e.g., non-parametric methods such as NPRS[116] or penalized likelihood methods[117], particularly for phylogenies with a low time depth, because it can allow for uncertainty in dates assigned to calibration points and does not require untested assumptions about the pattern of clock rate variation among lineages[118]. The greatest benefit of using a Bayesian method for dating is that the specification of prior distributions can be used to ensure that the analysis realistically incorporates the uncertainty associated with the calibration points used[119]. The procedure involves the user specifying both a phylogenetic model (a model of population history; the tree prior) and a clock model (of substitution rate variation); however, the likelihood calculation is based on the clock model only. In addition, as in any BM, one must specify a substitution model for each data partition. Rate variation between adjacent branches is assumed to be uncorrelated, as these rates did not show autocorrelation in recent studies[120]. BEAST can implement several combinations of tree and clock models; the present study used the following criteria to compare and choose tree prior and clock models.

Beginning with a Yule tree prior and an uncorrelated log-normal (relaxed) clock model, and with a prior on the tree height and mean substitution rate, each available model was evaluated. Firstly, any models estimating implausible TMRCAs on the outgroup were discounted. Implausible TMRCAs are, for example, those dated to the pre-Cambrian. For those models which gave stable results, the ratio of the marginal likelihoods (with respect to the prior) of alternative models (i.e., the Bayes Factor, BF) was used to choose between them[121] (here importance sampling and the harmonic mean of the sampled likelihoods are used as estimators of the marginal likelihoods). Where there was a tie between two models both were run and the model giving the higher ESS values (most efficient run) was chosen; there was no marked difference in parameter estimates for such ties. Choice of nucleotide substitution model followed that for the BM described above (based on MrModeltest), followed by use of BF tests to look for simpler, equally suitable, models for use in BEAST.

## Competing interests

The authors declare that they have no competing interests.

## Authors' contributions

LL Performed molecular work, parsimony analysis and wrote part of the Methods. MMHM, MAI, HSL, RPVJR, FS, JLD and ESU assisted with location, collection and identification of taxa and provided laboratory facilities and parasitological screening. SWA assisted with sample collection, collation, performed all remaining analyses and wrote the remaining sections of the paper. All authors read and approved the final manuscript.

## Supplementary Material

Additional file 1 ***Indoplanorbis exustus *adult specimen collected in Khon Kaen, Thailand**. Scale 1 cm. Click here for file

Additional file 2 **Phylogram representing a possible history for *Indoplanorbis *driven solely by geophysical events (changes in orogenics, eustasy, climate, etc.)**. This phylogeny was used as the test hypothesis in the SH-test. Click here for file

Additional file 3 **Matrix used in the initial sensitivity analysis for the parsimony method**. Horizontal axis, weighting matrix; vertical axis, clade. Clade status: M, monophyletic; PA, paraphyletic; PO, polyphyletic, on best tree found by tree search using the corresponding weighting scheme. Click here for file

Additional file 4 **Matrix of MRI values for different data partitioning schemes and character weighting regimes**. Click here for file
