# Supplementary figures and images for: The phylogeography of Indoplanorbis exustus (Gastropoda: Planorbidae) in Asia
Source: Parasit Vectors. 2010 Jul 5;3:57. doi: 10.1186/1756-3305-3-57 (PMC2914737; doi:10.1186/1756-3305-3-57)

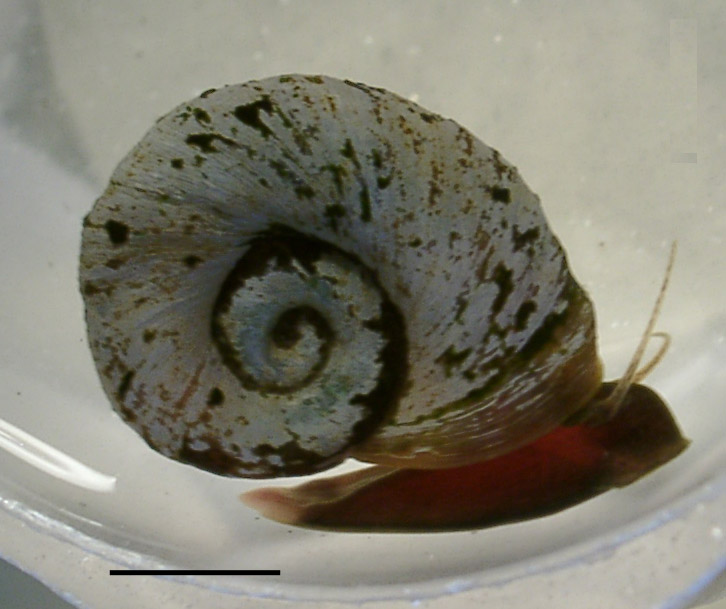

Supplement: Additional file 1 — Indoplanorbis exustus adult specimen collected in Khon Kaen, Thailand. Scale 1 cm. [file 1756-3305-3-57-S1.JPEG]

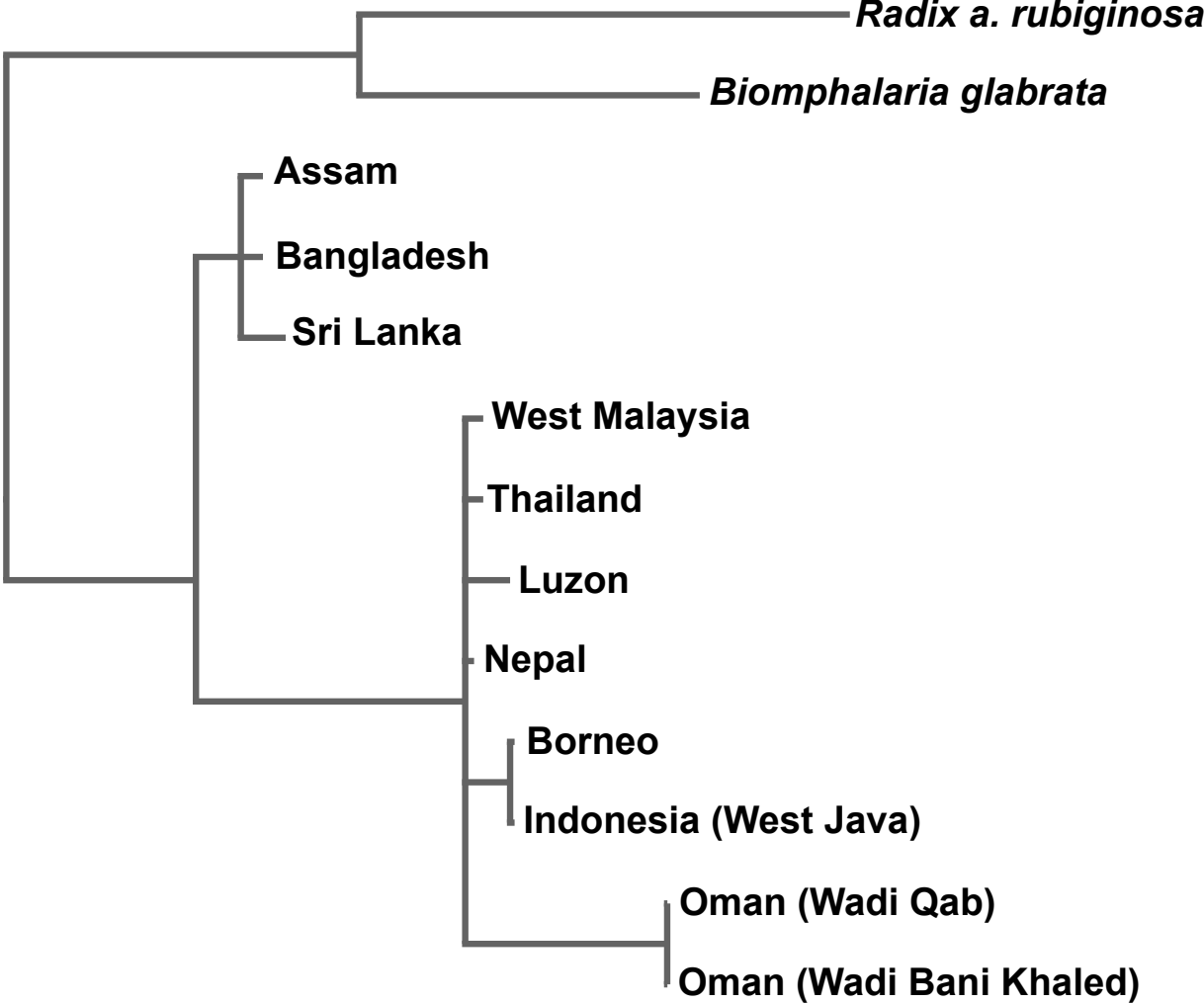

Supplement: Additional file 2 — Phylogram representing a possible history for Indoplanorbis driven solely by geophysical events (changes in orogenics, eustasy, climate, etc.). This phylogeny was used as the test hypothesis in the SH-test. [file 1756-3305-3-57-S2.PDF]
