# Supplementary material for: The phylogeography of Indoplanorbis exustus (Gastropoda: Planorbidae) in Asia
Source: Parasit Vectors. 2010 Jul 5;3:57. doi: 10.1186/1756-3305-3-57 (PMC2914737; doi:10.1186/1756-3305-3-57)
Supplement: Additional file 3 — Matrix used in the initial sensitivity analysis for the parsimony method. Horizontal axis, weighting matrix; vertical axis, clade. Clade status: M, monophyletic; PA, paraphyletic; PO, polyphyletic, on best tree found by tree search using the corresponding weighting scheme. [file 1756-3305-3-57-S3.PDF]

*rrn*L

111	211	411	811	1611	3211	6411	121	221	421	821	621	3221	6421	141	241	441	841	1641	3241	6441	181	281	481	881	1681	3281	6481	1161	2161	4161	8161	16161	32161	64161	1321	2321	4321	8321	16321	32321	64321	
M	M	M	M	PO	PO	PO	M	M	M	M	M	PO	PO	M	M	M	M	M	M	PO	M	M	M	M	M	M	M	M	M	M	M	M	M	M	M	M	M	M	M	PO	M	(3 4 5 6 7 10 11 12 13)
M	M	M	M	M	M	M	M	M	M	M	M	PO	M	PO	M	M	M	M	M	M	M	M	M	M	M	M	M	M	M	M	M	M	M	M	PO	PO	M	M	M	M	M	(3 4 5 6 7 11 12 13)
-	-	-	-	-	-	-	-	-	-	-	-	-	-	-	-	-	-	-	-	-	-	-	-	-	-	-	-	-	-	-	-	-	-	-	-	-	-	-	-	-	-	(5 6 7 10 11 13)
-	-	-	-	-	-	-	-	-	-	-	-	-	-	-	-	-	-	-	-	-	-	-	-	-	-	-	-	-	-	-	-	-	-	-	-	-	-	-	-	-	-	(7 10 11 13)
-	-	-	-	-	-	-	-	-	-	-	-	-	-	-	-	-	-	-	-	-	-	-	-	-	-	-	-	-	-	-	-	-	-	-	-	-	-	-	-	-	-	(3 4 12)
-	-	-	-	-	-	-	-	-	-	-	-	-	-	-	-	-	-	-	-	-	-	-	-	-	-	-	-	-	-	-	-	-	-	-	-	-	-	-	-	-	-	(7 11 13)
-	-	-	-	-	-	-	-	-	-	-	-	-	-	-	-	-	-	-	-	-	-	-	-	-	-	-	-	-	-	-	-	-	-	-	-	-	-	-	-	-	-	(3 4)
PO	PO	PO	PO	PO	PO	PO	PO	PO	PO	PO	PO	PO	PO	PO	PO	PO	PO	PO	PO	PO	PO	PO	PO	PO	PO	PO	PO	PO	PO	PO	PO	PO	PO	PO	PO	PO	PO	PO	PO	PO	PO	(5 6)
PA	PA	PA	PA	M	M	M	PA	PA	PA	PA	PA	M	M	PA	PA	PA	PA	PA	PA	M	PA	PA	PA	PA	PA	PA	PA	PA	PA	PA	PA	PA	PA	PA	PA	PA	PA	PA	PA	PA	PA	(8 9)

*cox*1

111	211	411	811	1611	3211	6411	121	221	421	821	621	3221	6421	141	241	441	841	1641	3241	6441	181	281	481	881	1681	3281	6481	1161	2161	4161	8161	16161	32161	64161	1321	2321	4321	8321	16321	32321	64321	
M	M	M	M	M	M	M	M	M	M	M	M	M	M	M	M	M	M	M	M	M	M	M	M	M	M	M	M	M	M	M	M	M	M	M	M	M	M	M	M	M	M	(3 4 5 6 7 10 11 12 13)
M	M	M	M	M	M	M	M	M	M	M	M	M	M	M	M	M	M	M	M	M	M	M	M	M	M	M	M	M	M	M	M	M	M	M	M	M	M	M	M	M	M	(3 45 6 7 11 12 13)
PO	PO	PO	PO	PO	PO	PO	PO	PO	PO	PO	PO	PO	PO	PO	PO	PO	PO	PO	PO	PO	PO	PO	PO	PO	PO	PO	PO	PO	PO	PO	PO	PO	PO	PO	PO	PO	PO	PO	PO	PO	PO	(5 6 7 10 11 13)
PO	PO	PO	PO	PO	PO	PO	PO	PO	PO	PO	PO	PO	PO	PO	PO	PO	PO	PO	PO	PO	PO	PO	PO	PO	PO	PO	PO	PO	PO	PO	PO	PO	PO	PO	PO	PO	PO	PO	PO	PO	PO	(7 10 11 13)
M	M	M	M	M	M	M	M	M	M	M	M	M	M	M	PO	M	M	M	M	M	M	M	M	M	M	M	M	M	M	PO	M	M	M	M	M	M	PO	PO	M	M	M	(3 4 12)
M	M	M	M	M	M	M	M	M	M	M	M	M	M	M	M	M	M	M	M	M	M	M	M	M	M	M	M	M	M	M	M	M	M	M	M	M	M	M	M	M	M	(7 11 13)
M	M	M	M	M	M	M	M	M	M	M	M	M	M	M	M	M	M	M	M	M	PO	M	M	M	M	M	M	M	M	M	M	M	M	M	M	M	M	M	M	M	M	(3 4)
M	M	M	M	M	M	M	M	M	M	M	M	M	M	M	M	M	M	M	M	M	M	M	M	M	M	M	M	M	M	M	M	M	M	M	M	M	M	M	M	M	M	(5 6)
M	M	M	M	M	M	M	M	M	M	M	M	M	M	M	M	M	M	M	M	M	M	M	M	M	M	M	M	M	M	M	M	M	M	M	M	M	M	M	M	M	M	(8 9)

*cox*1 1st&2nd Codons:

111	211	411	811	1611	3211	6411	121	221	421	821	621	3221	6421	141	241	441	841	1641	3241	6441	181	281	481	881	1681	3281	6481	1161	2161	4161	8161	16161	32161	64161	1321	2321	4321	8321	16321	32321	64321	
PO	PO	PO	PO	PO	PO	PO	M	M	M	M	M	M	M	PO	M	M	M	M	M	M	PO	M	M	M	M	M	M	PO	M	M	M	M	M	M	PO	M	M	M	M	M	M	(3 45 67 10 11 1213)
PO	PO	PO	PO	PO	PO	PO	PO	PO	PO	PO	PO	PO	PO	PO	PO	PO	PO	PO	PO	PO	PO	PO	PO	PO	PO	PO	PO	PO	PO	PO	PO	PO	PO	PO	PO	PO	PO	PO	PO	PO	PO	(3 45 6 7 11 12 13)
PO	PO	PO	PO	PO	PO	PO	PO	PO	PO	PO	PO	PO	PO	PO	PO	PO	PO	PO	PO	PO	PO	PO	PO	PO	PO	PO	PO	PO	PO	PO	PO	PO	PO	PO	PO	PO	PO	PO	PO	PO	PO	(5 6 7 10 11 13)
PO	PO	PO	PO	PO	PO	PO	PO	PO	PO	PO	PO	PO	PO	PO	PO	PO	PO	PO	PO	PO	PO	PO	PO	PO	PO	PO	PO	PO	PO	PO	PO	PO	PO	PO	PO	PO	PO	PO	PO	PO	PO	(7 10 11 13)
PO	PO	PO	PO	PO	PO	PO	PO	PO	PO	PO	PO	PO	PO	PO	PO	PO	PO	PO	PO	PO	PO	PO	PO	PO	PO	PO	PO	PO	PO	PO	PO	PO	PO	PO	PO	PO	PO	PO	PO	PO	PO	(3 4 12)
M	M	M	M	M	M	M	M	M	M	M	M	M	M	M	M	M	M	M	M	M	M	M	M	M	M	M	M	M	M	M	M	M	M	M	M	M	M	M	M	M	M	(7 11 13)
PO	PO	PO	PO	PO	PO	PO	PO	PO	PO	PO	PO	PO	PO	PO	PO	PO	PO	PO	PO	PO	PO	PO	PO	PO	PO	PO	PO	PO	PO	PO	PO	PO	PO	PO	PO	PO	PO	PO	PO	PO	PO	(3 4)
PO	PO	PO	PO	PO	PO	PO	PO	PO	PO	PO	PO	PO	PO	PO	PO	PO	PO	PO	PO	PO	PO	PO	PO	PO	PO	PO	PO	PO	PO	PO	PO	PO	PO	PO	PO	PO	PO	PO	PO	PO	PO	(5 6)
M	M	M	M	M	M	M	M	M	M	M	M	M	M	M	M	M	M	M	M	M	M	M	M	M	M	M	M	M	M	M	M	M	M	M	M	M	M	M	M	M	M	(8 9)

*cox*1 3rd Codons:

111	211	411	811	1611	3211	6411	121	221	421	821	621	3221	6421	141	241	441	841	1641	3241	6441	181	281	481	881	1681	3281	6481	1161	2161	4161	8161	16161	32161	64161	1321	2321	4321	8321	16321	32321	64321	
M	M	M	M	M	M	M	M	M	M	M	M	M	M	M	M	M	M	M	M	M	M	M	M	M	M	M	M	M	M	M	M	M	PO	PO	M	M	M	M	M	M	PO	(3 4 5 6 7 10 11 12 13)
M	M	M	M	M	M	M	M	M	M	M	M	M	M	M	M	M	M	M	M	M	M	M	M	M	M	M	M	M	M	M	M	M	M	M	M	M	M	M	M	M	M	(3 4 5 6 7 11 12 13)
PO	PO	PO	PO	PO	PO	PO	PO	PO	PO	PO	PO	PO	PO	PO	PO	PO	PO	PO	PO	PO	PO	PO	PO	PO	PO	PO	PO	PO	PO	PO	PO	PO	PO	PO	PO	PO	PO	PO	PO	PO	PO	(5 6 7 10 11 13)
PO	PO	PO	PO	PO	PO	PO	PO	PO	PO	PO	PO	PO	PO	PO	PO	PO	PO	PO	PO	PO	PO	PO	PO	PO	PO	PO	PO	PO	PO	PO	PO	PO	PO	PO	PO	PO	PO	PO	PO	PO	PO	(7 10 11 13)
M	M	M	M	M	M	M	PO	M	M	M	M	M	M	PA	M	M	M	M	M	M	PO	M	M	M	M	M	M	PO	PO	M	M	M	PO	PO	PO	PO	M	M	M	M	PO	(3 4 12)
M	M	M	M	M	M	M	M	M	M	M	M	M	M	M	M	M	M	M	M	M	M	M	M	M	M	M	M	M	M	M	M	M	M	M	M	M	M	M	M	M	M	(7 11 13)
M	M	M	M	M	M	M	PO	M	M	M	M	M	M	PO	M	M	M	M	M	M	PO	M	M	M	M	M	M	PO	PO	M	M	M	PO	PO	PO	PO	M	M	M	M	PO	(3 4)
M	M	M	M	M	M	M	M	M	M	M	M	M	M	M	M	M	M	M	M	M	M	M	M	M	M	M	M	M	M	M	M	M	M	M	M	M	M	M	M	M	M	(5 6)
M	M	M	M	M	M	M	M	M	M	M	M	M	M	M	M	M	M	M	M	M	M	M	M	M	M	M	M	M	M	M	M	M	M	M	M	M	M	M	M	M	M	(8 9)

*cox*1+*rrn*L:

111	211	411	811	1611	3211	6411	121	221	421	821	1621	3221	6421	141	241	441	841	1641	3241	6441	181	281	481	881	1681	3281	6481	1161	2161	4161	8161	16161	32161	64161	1321	2321	4321	8321	16321	32321	64321	
PO	PA	PA	PA	PO	PO	PO	PA	PA	PA	PA	PA	PO	PO	PA	PA	PA	PA	PA	PA	PO	PA	PA	PA	PA	PA	PA	PA	PA	PA	PA	PA	PA	PA	PA	PA	PA	PA	PA	PA	PA	PA	(3 4 5 6 7 10 11 12 13)
PO	PA	PA	PA	PA	PA	PA	PA	PA	PA	PA	PA	PA	PA	PA	PA	PA	PA	PA	PA	PA	PA	PA	PA	PA	PA	PA	PA	PA	PA	PA	PA	PA	PA	PA	PA	PA	PA	PA	PA	PA	PA	(3 4 5 6 7 11 12 13)
PO	PO	PO	PO	PO	PO	PO	PO	PO	PO	PO	PO	PO	PO	PO	PO	PO	PO	PO	PO	PO	PO	PO	PO	PO	PO	PO	PO	PO	PO	PO	PO	PO	PO	PO	PO	PO	PO	PO	PO	PO	PO	(5 6 7 10 11 13)
PO	PO	PO	PO	PO	PO	PO	PO	PO	PO	PO	PO	PO	PO	PO	PO	PO	PO	PO	PO	PO	PO	PO	PO	PO	PO	PO	PO	PO	PO	PO	PO	PO	PO	PO	PO	PO	PO	PO	PO	PO	PO	(7 10 11 13)
PO	PA	PA	PA	PO	PO	PO	PO	PA	PA	PA	PA	PO	PO	PA	PA	PA	PA	PA	PA	PO	PA	PA	PA	PA	PA	PA	PA	PA	PO	PA	PA	PA	PA	PA	PA	PA	PA	PA	PO	PA	PA	(3 4 12)
M	M	M	M	M	M	M	M	M	M	M	M	M	M	M	M	M	M	M	M	M	M	M	M	M	M	M	M	M	M	M	M	M	M	M	M	M	M	M	M	M	M	(7 11 13)
PO	PO	PO	PO	PO	PO	PO	PO	PO	PO	PO	PO	PO	PO	PO	PO	PO	PO	PO	PO	PO	PO	PO	PO	PO	PO	PO	PO	PO	PO	PO	PO	PO	PO	PO	PO	PO	PO	PO	PO	PO	PO	(3 4)
M	M	M	M	M	M	M	M	M	M	M	M	M	M	M	PO	M	M	M	M	M	M	M	M	M	M	M	M	M	M	M	M	M	M	M	M	M	M	M	M	M	M	(5 6)
M	M	M	PA	M	M	M	M	M	M	M	PA	M	M	M	M	M	M	PA	PA	M	M	M	M	M	PA	PA	PA	M	M	M	M	PA	PA	PA	M	M	M	M	M	M	PA	(8 9)

Taxa:

1 *Radix auricularia rubiginosa*
2 *Biomphalaria glabrata*
3 Oman (Wadi Qab)
4 Oman (Wadi Bani Khaled)
5 Indonesia (West Java)
6 Borneo
7 Thailand (Khon Kaen)
8 Assam
9 Bangladesh
10 Sri Lanka
11 Luzon,
12 West Malaysia
13 Nepal
